# Time is of the essence: using archived samples in the development of a GT-seq panel to preserve continuity of ongoing genetic monitoring

**DOI:** 10.7717/peerj.20726

**Published:** 2026-02-04

**Authors:** Guilherme Caeiro-Dias, Megan J. Osborne, Thomas F. Turner

**Affiliations:** Department of Biology and Museum of Southwestern Biology, University of New Mexico, Albuquerque, New Mexico, United States

**Keywords:** Temporal genetic variation, Targeted amplicon sequencing, Microhaplotypes, SNPs

## Abstract

Genotyping-in-Thousands by sequencing (GT-seq) is a promising tool for genetic monitoring. For the past 25 years, genetic monitoring of Rio Grande silvery minnow (*Hybognathus amarus*) has been conducted annually by surveying variation at microsatellite loci. This is the first study describing the development of a GT-seq panel using archived samples that maintains the analytical and inferential continuity of long-term genetic monitoring. A total of 2,983 microhaplotypes in 373 individuals were identified using nextRAD-seq from samples spanning 20 years and a conspecific reference genome. Using this data, estimates of genetic diversity and temporal genetic structure across the time-series were used as a baseline to test subsets of loci that effectively tracked those changes. A panel including 250 loci with higher F_ST_ across temporal samples and 250 loci selected randomly offered the highest power and was used for GT-seq optimization. A sex-linked marker validated previously was also included for sex assignment. The optimized GT-seq panel included 284 loci. Comparisons of genotypes from those loci obtained for the same samples with nextRAD-seq and GT-seq revealed high genotype accuracy (98.3%). Estimates of genetic diversity and patterns of temporal genetic structure were similar between datasets and accuracy of sex assignment was 100%. The utility of using a conspecific genome for both loci identification and primer design in the face of reduced genetic diversity, and the importance of temporal metrics representative of ongoing genetic monitoring is explored. The strategy used here, effectively preserved the long-term genetic monitoring of the endangered Rio Grande silvery minnow while transitioning to a more efficient and cost-effective marker system.

## Introduction

Single nucleotide polymorphisms (SNPs) are now extensively used in the fields of ecology and conservation, including population genomics (Hohenlohe et al., 2010), parentage analysis (Tokarska et al., 2009), population assignment (Camak, Osborne & Turner, 2021), detection of adaptation (Narum & Hess, 2011), and introgression (Caeiro-Dias et al., 2023a). Genome-wide representation sequencing methods allows cost effective genotyping of thousands of SNPs across several hundred individuals and permits fine-scale genetic patterns to be investigated because additional loci increase resolution compared to microsatellites (Liu et al., 2005; Hess, Matala & Narum, 2011). In many cases, detection of admixture is more accurate with SNPs than with microsatellites (Szatmári et al., 2021; Miralles et al., 2024). These characteristics make genome-wide SNPs and SNP-based markers useful molecular tools for genetic monitoring because they allow reliable evaluation of temporal shifts of genomic diversity and accurate estimation of effective population size (N_e_) (Clark et al., 2025), and can be used for stock assignment (Gilbey et al., 2016; McKinney, Seeb & Seeb, 2017), monitoring introgression and hybridization (Andersen et al., 2016; Henriques et al., 2018; Chang et al., 2022), evaluating relatedness within populations (Roques et al., 2019), and for estimating demographic parameters from parentage-based tagging methods (Trenkel et al., 2022).

In studies involving genetic monitoring it is necessary to genotype many samples (hundreds to thousands) across multiple generations. In these cases, costs of reduced-representation sequencing methods may become prohibitive for most research projects with limited resources. Moreover, genetic monitoring quantifies temporal changes in population genetic diversity, provides estimates of benchmark metrics over contemporary timescales, and provides insights into demographic and evolutionary processes, such as adaptive potential, population growth, and extinction risk (Schwartz, Luikart & Waples, 2007). It is thus critical that the same genomic regions are consistently monitored across time. Amplicon-targeted methods provide two main advantages for genetic monitoring compared to genome-wide reduced-representation sequencing methods. It is more economical and offers a way to consistently monitor the same genomic regions.

Genotyping-in-Thousands by sequencing (GT-seq) is a method of targeted SNP genotyping that uses multiplexed polymerase chain reaction (PCR) amplicon sequencing (Campbell, Harmon & Narum, 2015) that allows simultaneous amplification of hundreds of targeted loci referred to as a GT-seq panel. Once a GT-seq panel is developed and optimized for the focal species, it provides an efficient means of monitoring genetic variation and effective population size (N_e_) across hundreds of genetic loci collected over contemporary timescales. GT-seq panels have been developed and applied in vertebrate species including salmon (May et al., 2020; McKinney et al., 2020; Willis et al., 2021), walleye (*Sander vitreus*, Bootsma et al., 2020), western rattlesnake (*Crotalus oreganus*, Schmidt et al., 2020), and northern Idaho ground squirrel (*Urocitellus brunneus*, Garrett et al., 2024). Panels have been developed for a single or multiple purposes including parentage analysis, relatedness, individual identification, monitoring of hybridization, spatial population structure analysis and genetic stock identification (Campbell et al., 2019; Chang et al., 2022; Harris et al., 2024; Hayward et al., 2022; Le Gall et al., 2024). Sex-specific markers can also be included on GT-seq panels to aid in parentage analysis (Burgess, Irvine & Russello, 2022; Hayward et al., 2022). Moreover, individual sex identification at the population level allows estimation of sex ratios where sex cannot be determined phenotypically. This is important because variation in sex ratio across space and time is a fundamental driver of population dynamics (Ancona et al., 2017). From a conservation perspective, biased sex ratio can heighten extinction risk (Wedekind, 2012) and result in greater rates of genetic drift because N_e_ is reduced below its theoretical maximum (Wright, 1938).

Rio Grande silvery minnow (*Hybognathus amarus*) is a small-bodied (<90 mm standard length), short-lived (1–3 years; Horwitz et al., 2018) member of the Family Leuciscidae. This species was historically widely distributed in the Rio Grande from northern New Mexico to the Gulf of Mexico, and in the Pecos River from northern New Mexico to the confluence of the Rio Grande in Texas (Pflieger, 1980). Flow and habitat changes associated with dams and diversion structures resulted in significant range contraction (Bestgen & Platania, 1991; Mortensen et al., 2024; Turner et al., 2015). The interaction of these changes with species life-history causes changes in population density that span several orders of magnitude from one year to the next (Dudley, Platania & Mortensen, 2024). Today, a remnant population persists in about 280-km reach of the Rio Grande in New Mexico, constituting less than 95% of the original range (Bestgen & Platania, 1991). The species was listed as endangered in 1994 (U.S. Fish and Wildlife Service, 1994).

For the past 25 years, genetic monitoring of Rio Grande silvery minnow has been conducted annually providing key information for species management (Osborne et al., 2024). The monitoring program began using nine microsatellite loci (Alò & Turner, 2005; Osborne, Carson & Turner, 2012) and recently a temporal genome-wide microhaplotype (3,151 loci containing 5,549 microhaplotyped SNPs) dataset was obtained, spanning 20 years (Osborne, Caeiro-Dias & Turner, 2023). A side-by-side comparison of datasets showed that some estimates of genetic diversity and N_e_ had smaller confidence intervals (CIs) when including genome-wide microhaplotypes, even with smaller sample sizes used (Osborne, Caeiro-Dias & Turner, 2023). Although broad trends and biological inferences were similar between datasets, some metrics were not consistent across marker types (*e.g*., allelic richness, linkage disequilibrium N_e_). Furthermore, significant temporal changes in allele frequencies observed in the microhaplotype dataset were consistent with the microsatellite dataset (Osborne, Caeiro-Dias & Turner, 2023; Osborne et al., 2024). This phenomenon was most obvious after a multi-year population bottleneck (2013–2014). In another study, one locus with two sex-associated SNPs was identified and validated (Caeiro-Dias et al., 2023b) permitting the sex of individuals to be identified genetically.

Some studies have utilized previously developed GT-seq panels to genotype archived samples (Arpin et al., 2024; Setzke, Wong & Russello, 2021), while others have developed panels to complement on-going population monitoring (Hayward et al., 2022). The present study is the first to our knowledge, that used only archived collections (either DNA or tissue) to develop a GT-seq panel. This allowed explicit design of a panel of markers to maintain the analytical and inferential continuity of long-term genetic monitoring. The goals of this study were to (i) identify a set of loci that could be readily screened using the GT-seq protocol (Campbell, Harmon & Narum, 2015) from microhaplotype genome-wide time-series data and that included the sex-specific marker, (ii) ensure that the panel tracked genetic diversity and temporal variation detected in previous genetic monitoring, and (iii) validate the panel by comparing the results with those obtained from microhaplotype genome-wide data using the same samples.

## Methods

### Draft reference genome: DNA extraction, sequencing, and assembly

A muscle tissue sample from a freshly collected (4 November 2016) Rio Grande silvery minnow was used for whole-genome sequencing. High molecular weight genomic DNA was obtained with a standard phenol-chloroform DNA extraction. The quantity of DNA was determined using Qubit (Thermo Fisher Scientific, Waltham, MA, USA). Genomic DNA (1,000 ng total) was adjusted to 50 μl in 10 nM Tris-HCl + 0.1 nM EDTA (pH 8.0), sheared on a Covaris M220 instrument using microTUBE-50, and specifying the ‘DNA-550-bp’ parameter. Sheared DNA was then used to generate a genomic library using a KAPA HyperPrep Kit (KAPA Biosystems, Wilmington, MA, USA) following manufacturer’s protocol. Briefly, the protocol has five steps: (i) end repair and A-tailing; (ii) ligation of a barcoded adapter (compatible with Illumina’s sequencing kits for NextSeq 500 platform); (iii) post-ligation bead clean-up; (iv) library amplification; and (v) post-amplification bead cleanup. Library size selection was performed on a BluePippin (Sage Science, Massachusetts, MA, USA) apparatus with a target fragment size of 550 bp. Library quality, quantity and fragment size distribution were assessed using Qubit and Bioanalyzer. The library was further quantified using a KAPA Library Quantification Kit for the Illumina platform (KAPA Biosystems, Wilmington, MA, USA) following manufacturer’s protocol and then sequenced on an Illumina NextSeq 500 instrument to obtain 200 bp paired-end reads.

Sequenced reads were trimmed with Trimmomatic v. 0.36 (Bolger, Lohse & Usadel, 2014) to remove low quality bases. Bases on both extremes of each read were removed if quality was below 25 or if an ambiguous (N) base call was observed. Each read was then scanned with a 2-base wide sliding window, cutting when the average quality per base dropped below 25. After trimming, reads were discarded if smaller than 36 bp. Retained trimmed reads were used for the draft genome assembly in MEGAHIT v. 1.1.3 (Li et al., 2015, 2016) using default parameters. Genome assembly statistics were assessed with gVolante v. 2.0.0 (https://gvolante.riken.jp/; Nishimura, Hara & Kuraku, 2017).

### Microhaplotype identification and primer design

**Figure 1:**
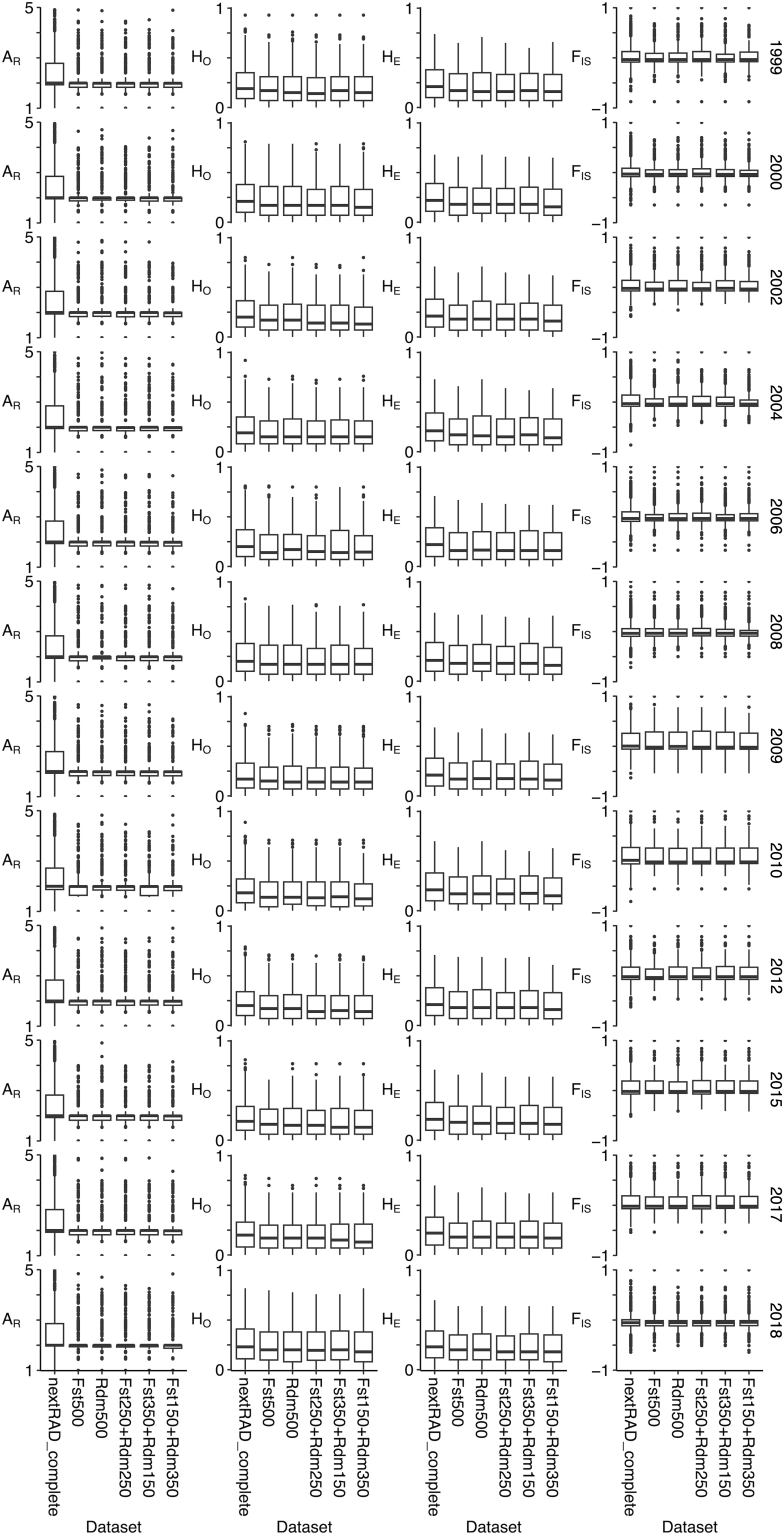
Summary statistics of genetic diversity estimated with the five subsets of 500 loci used to compare with results from nextRAD_complete dataset to identify a set of markers that tracked temporal changes in genetic diversity. From left to right, each column correspond to allelic richness (A_R_), observed heterozygosity (H_O_), expected heterozygosity (H_E_), and inbreeding coefficient (F_IS_) were estimated for each temporal collection (identified on the right) using the nextRAD_complete dataset (2,983 loci) and five subsets of 500 loci with GT-seq primers designed (loci with higher F_ST_ (F_ST_500), randomly selected loci (Rdm500), and different combinations of “F_ST_” plus “Random” (F_ST_350+Rdm150, F_ST_250+Rdm250, F_ST_150+Rdm350)).

Genome-wide SNP identification using Nextera-tagmented reductively-amplified DNA sequencing (nextRAD-seq) as described by Russello et al. (2015) was performed with the data from 379 individuals reported in Osborne, Caeiro-Dias & Turner (2023), comprising 12 temporal collections that spanned 20 years and the entire remnant distribution of this species (a distribution map depicting current range can be found in Fig. 1a in Osborne, Caeiro-Dias & Turner, 2023). This dataset bracketed population bottlenecks previously identified in demographic (Yackulic et al., 2022) and genetic data (Osborne, Caeiro-Dias & Turner, 2023; Osborne et al., 2024). Archived samples held in the Museum of Southwestern Biology Division of Fishes were selected based on preservation method, year of collection, and collection locality. Sample selection and assessment of sample quality is described in Osborne, Caeiro-Dias & Turner (2023). In short, genomic data was obtained from three different sampling preservation methods. Earlier collections included frozen tissue preserved at −80 °C, and purified DNA from previous DNA isolated with phenol chloroform while tissue from more recent collections were preserved in 95% ethanol. The three methods yielded similar amounts of genomic data although data from frozen tissue and purified DNA was slightly higher. Microhaplotypes were identified using methods described in Osborne, Caeiro-Dias & Turner (2023), but with four modifications: nextRAD loci were identified using the draft genome reported here as reference to maximize the number of SNPs identified (the first two genomes made available to date (Caeiro-Dias et al., 2023a) had not been sequenced at this stage of the present study); no depth of coverage filter was applied to nextRAD loci before variant calling; loci were discarded if mean depth of coverage was lower than 20; and only individuals with less than 25% missing data were retained. Bioinformatic details on microhaplotype identification from nextRAD data are provided in Table S1. To retain a set of putative neutral loci, those deviating from Hardy-Weinberg equilibrium (HWE) and in linkage disequilibrium (LD) across the time-series were discarded. Departures from HWE were tested using a classic chi-square test on haplotyped data with R package pegas v. 1.0 (Paradis, 2010) and using the Bonferroni correction for multiple comparisons implemented in the R package rcompanion v. 2.3.21 (Mangiafico, 2020) as implemented in the R function multi_HWE_tests v. 1.0.0 (https://github.com/gcaeirodias/multi_HWE_tests). Loci were considered as deviating from HWE if tests were significant (*p*-value < 0.05) across all 12 temporal collections. Estimations of LD were performed on SNP data using one SNP randomly selected per locus. If a SNP in LD was removed, then the entire locus was removed. The coefficient of correlation r^2^ was estimated with the R package GUSLD v. 1.0.1 (Bilton et al., 2018). Tests for LD were performed using chi-square tests with Bonferroni corrections to account for multiple simultaneous tests as implemented in the R pipeline significantLD v. 1.0.0 (https://github.com/gcaeirodias/significantLD). Microhaplotypes and individuals retained after all filtering steps are hereafter referred to as nextRAD_complete dataset. Although this dataset included biallelic SNPs and microhaplotypes, for simplicity only the term “microhaplotypes” is used when refering to those loci.

To facilitate primer design and maximize the number of retained loci, SNPs were filtered out based on their position on the nextRAD locus sequence. If a SNP was found within the first or last 33 bp, all positions from the beginning (or end) of the locus to the SNP were discarded. The loci were again screened for SNPs within the next 33 bp region starting in the first position after the discarded SNP. The process was repeated iteratively such that each nextRAD locus had at least 33 bp before the first and after the last SNP retained. The first and last 25 bp allowed sufficient flanking regions for primer design while the other 8 bp were used to design *in silico* probes for GT-seq genotyping, ensuring that potential primers and probes were not overlapping. Loci that resulted in sequences longer than 150 bp were removed because they exceeded the length permitted by our sequencing approach. Those filtering steps were performed with the custom bash pipeline GT-seq_filters v. 1.0.0 developed for this study (https://github.com/gcaeirodias/GT-seq_filters). The software Primer3 command line v. 2.5.0 (Untergasser et al., 2012) was used to design up to 5 primer pairs for each locus using the regions from Rio Grande silvery minnow draft genome corresponding to filtered loci as the template. Primer design parameters from Bootsma et al. (2020) were used with two modifications: primer lengths of 18 to 25 bp and product size of 100 to 150 bp were specified. The best primer pair designed for each locus was then mapped to the Rio Grande silvery minnow draft genome using the *blastn* program (Altschul et al., 1990) with the *blastn-short* task implemented in BLAST+ v. 2.9.0 (Camacho et al., 2009). If at least one primer matched one or more off-target sites with 100% coverage and identity, that primer pair was discarded. In those instances, the next best pair was mapped on the draft genome as previously described and the process was repeated until a primer pair mapped only to the target locus or until no primer pairs remained. In the latter case, the locus was discarded.

### Microhaplotype selection for GT-seq

Previous research has shown that up to 300 amplicons is sufficient to optimize PCR multiplex performance (Beacham et al., 2018; McKinney et al., 2018) and that choosing loci with greater genetic differentiation (*e.g*., F_ST_) maximizes accuracy for population genetic structure analysis (Ackerman, Habicht & Seeb, 2011; Storer et al., 2012). Our goal was to identify a subset of about 300 loci that tracked temporal changes in genetic variation. As such, the retained SNPs within a locus with primers successfully designed were haplotyped with rad_haplotyper.pl script (Willis et al., 2017) using the default settings for downstream analysis. Locus-specific F_ST_ across generations was then estimated using microhaplotype data and used as a criterion to select a dataset for GT-seq PCR multiplex optimization, as described below. Locus-specific F_ST_ values were used with the goal of selecting loci that were able to track temporal shifts, not divergent loci as typically used (*e.g*., Ackerman, Habicht & Seeb, 2011; Harris et al., 2024; Hayward et al., 2022; Storer et al., 2012).

After preliminary analysis using different combinations of loci, five subsets with higher F_ST_ and/or randomly selected loci were tested. In subsets with loci selected based on F_ST_, the remnant loci randomly selected were selected after excluding those with higher F_ST_ already selected to obtain a total of 500. One subset had 500 loci with higher F_ST_ (F_ST_500), and another had 500 randomly selected loci (Rdm500). The other three subsets were different combinations of “F_ST_” plus “Random”: F_ST_350+Rdm150, F_ST_250+Rdm250, and F_ST_150+Rdm350. To select one of those subsets of 500 loci for PCR multiplex optimization, allelic richness (A_R_), expected (H_E_) and observed heterozygosity (H_O_), inbreeding coefficient (F_IS_), and population-level pairwise F_ST_ between all years (*i.e*., temporal pairwise F_ST_) were estimated using the nextRAD_complete dataset and compared to each subset of 500 loci. Temporal pairwise F_ST_ and its significance were estimated using 10,000 permutations implemented in GenoDive v. 3.0.6 (Meirmans, 2020). All other metrics were estimated with the package diveRsity v. 1.9.90 (Keenan et al., 2013) in R; this and the following analyses conducted in R were performed in R v. 4.2.1 (R Core Team, 2022) in RStudio 2022.7.2.576 (Posit team, 2022).

Discriminant analyses of principal components (DAPC) were performed using the R package adegenet v. 1.3-1 (Jombart, 2008; Jombart & Ahmed, 2011) to compare multivariate coordinates for each year between the complete dataset and each data subset. DAPC summarizes genotypes in principal components (PCs) that are used to construct discriminant functions (DFs) that maximize among-group variation while minimizing within-group variation. Missing data (MD) was replaced within each group using the Breiman’s regression random forest algorithm (Breiman, 2001) implemented in R package randomForest v. 4.6–14 (Liaw & Wiener, 2002). Values of MD were predicted from 1,000 independently constructed regression trees and 100 bootstrap iterations with default bootstrap sample size. An initial DAPC was performed using years as groups, without scaling allele frequencies, retaining all PCs and DFs and keeping other options as default. The *a-score* method was used to select the optimal number of PCs retained. The final DAPC was performed using the optimal number of PCs, two DFs, and retaining the other default options.

Using the group (*i.e*., year) coordinates obtained from each subset the Euclidian distances (*d*) to the corresponding group coordinate from the complete dataset were estimated. The subset with results closest to the complete dataset was considered the one with smaller average of Euclidean distances.

### GT-seq library preparation and PCR multiplex optimization

After selecting a dataset of 500 loci, an initial double-indexed library was prepared to test the PCR multiplex efficacy to amplify the target loci. This library contained 88 samples used for nextRAD sequencing and SNP discovery (Table S2; also see Osborne, Caeiro-Dias & Turner, 2023) with sufficient DNA remaining for library construction. The same samples were used to track genotype accuracy (see below). Library preparation and sequencing were performed following Campbell, Harmon & Narum (2015) with two modifications. The read 1 primer (that allows sequencing of our target fragment), was used without the last adenine base (A); and single-end sequencing producing 150 bp reads was performed on an Illumina^®^ NextSeq 500. To facilitate sequencing, a custom index 2 primer was designed to read the i5 index (the reverse-complement of the read 1 primer). Demultiplexing was performed with BCLConvert v. 4.2.7 (Illumina^®^, Inc., San Diego, CA,USA; https://emea.support.illumina.com/sequencing/sequencing_software/bcl-convert.html) allowing one mismatch per index.

Demultiplexed data were used to estimate the number of reads containing the expected primer combination and the number of reads resulting from several types of primer interactions using the script *GTseq_Primer-Interactions.pl* from GTseq-Pipeline (https://github.com/GTseq/GTseq-Pipeline; Campbell, Harmon & Narum, 2015). The primary goal was to identify and remove primer pairs that disproportionally contributed to primer interactions. This increased depth of coverage of target loci with reduced number of primer interactions. Primers with excessive interactions with multiple primers from other pairs were discarded from PCR multiplex before preparing subsequent genomic libraries. In cases where a primer interacted mostly with one other primer, the pair that sequenced the locus with higher locus-specific F_ST_ across time-series was retained. Next, the GTscore pipeline v. 1.3 (https://github.com/gjmckinney/GTscore) was used to identify genotypes. Prior to the analysis of the first sequencing run results, in-silico probes were designed for each SNP to include eight nucleotides flanking for each SNP and to include variants when overlapping identified SNPs (see manual for details on probe design https://github.com/gjmckinney/GTscore/blob/master/GTScoreDocumentation%20V1.3.docx). *AmpliconRadCounter.pl* script was used to count the number of unique reads per individual, to identify on-target reads, and to count the number of reads containing each SNP allele for every individual. Then counts of reads containing a SNP allele for each individual were used for microhaplotype genotyping with the maximum likelihood algorithm described by McKinney et al. (2018) and implemented in *GTscore.R* script. Genotype accuracy between nextRAD_complete and GT-seq was estimated from samples genotyped for at least 70% of the loci.

The average proportion of primer interactions and average genotype accuracy previously estimated were used as PCR multiplex optimization criteria. A high proportion of reads resulting from primer interactions are expected to have a large negative impact on PCR multiplex performance. As such, the exclusion of primer pairs producing a high proportion of primer interactions have been applied to the optimization of GT-seq panels (Hayward et al., 2022; Schmidt et al., 2020). Another criterion commonly used to decide whether to exclude or keep loci in GT-seq panels is genotype accuracy (Bootsma et al., 2020; Schmidt et al., 2020).

The optimization process (library preparation, sequencing, evaluation of primer interactions, and genotype accuracy estimation) was repeated four times, until the proportion of reads from primer interactions was lower than one third of the sum of total reads and genotype accuracy higher than 95%. For the third and fourth rounds of optimization the sex-linked locus (HAM6) containing two SNPs, identified and validated by Caeiro-Dias et al. (2023a), was also included in the library preparation. If sex marker primers were involved in excessive primer interactions, the other primers were excluded. For the third optimization round, the overall genotype accuracy was high (see Results) but to further increase PCR multiplex performance, over and under-represented loci were removed by discarding loci with disproportional high or low numbers of reads. SNPs with genotype accuracy lower than 0.9 were also removed from the GT-seq panel, but in these cases, only SNPs were discarded, and loci were kept if they contained other SNPs that exceeded the threshold. Determinations of locus representation and SNP genotype accuracy were made based on individuals with less than 30% missing data. The final (fourth) optimization run was performed to confirm that the proportion of primer interactions was low, and genotype accuracy was high.

### GT-seq panel validation

The optimized GT-seq panel was used to prepare a library that included 30 samples from 1999 used for nextRAD-seq and 15 samples previously used to validate the HAM6 sex-marker (see details in Caeiro-Dias et al., 2023a). In that study, the sex-marker was validated with 100% accuracy using 95 females and 95 males identified phenotypically. The library was sequenced on an Illumina NextSeq 2000 with the custom index 2 primer described before to obtain 150 bp paired-end reads. Demultiplexing was performed as described above. Only read 1 was used for downstream analysis. In total, 118 samples (data from the 88 samples sequenced in the fourth optimization round and additional 30 samples from 1999) were sequenced with both nextRAD-seq and GT-seq approaches (Table 1) to validate the utility of the GT-seq_283 to obtain results comparable to the complete dataset. The other 15 samples previously sequenced for HAM6 were used to evaluate the performance of the sex-linked marker primers in the GT-seq panel. Some of the samples had large amounts of missing data (results not shown), and the entire process was repeated (library preparation and sequencing) for all the 133 samples to increase depth of coverage and ensure that all loci were sequenced with sufficient depth of coverage across the majority of the samples. Allele reads were counted with *AmpliconRadCounter.pl* script and the *GTscore.R* script was run to identify microhaplotypes. Individuals with missing data higher than 30% were removed.

**Table 1:** Number of archived samples used from each collection and samples sizes in each dataset. Total number of samples (*N*) used to prepare nextRAD libraries, the number of samples retained after filtering to obtain the complete set of 2,983 loci comprising 5,315 SNPs (nextRAD_complete), the number of samples genotyped with the optimized GT-seq panel (GT-seq_283) with less than 30% missing data and that were shared with nextRAD_complete (nextRAD_72ind). Missing data for each year in each dataset is shown on the corresponding column on the right (MD). For GT-seq_283, the number of samples and MD in parentheses correspond to those retained but not used for GT-seq panel validation due to small sample sizes.

Year	*N*	nextRAD_complete	nextRAD_complete (MD)	GT-seq_283	GT-seq_283 (MD)	nextRAD_72ind	nextRAD_72ind (MD)
1999	30	28	0.06	21	0.1	21	0.05
2000	42	42	0.02	0	–	0	–
2002	30	30	0.01	14	0.004	14	0.01
2004	27	26	0.01	10	0.03	10	0.01
2006	30	30	0.04	0	–	0	–
2008	30	30	0.02	0 (6)	– (0.09)	0	–
2009	30	29	0.05	0	–	0	–
2010	29	28	0.08	0	–	0	–
2012	30	30	0.02	0 (9)	– (0.05)	0	–
2015	31	31	0.02	0 (7)	– (0.04)	0	–
2017	30	30	0.03	11	0.01	11	0.02
2018	40	39	0.02	16	0.03	16	0.02
Total	379	373	0.03	72 (22)	0.03 (0.05)	72	0.02

Next, using the GT-seq_283 dataset for all years with 10 or more samples (see Table 1 for details on samples retained and sample size per year) A_R_, H_O_, H_E_, F_IS_, and pairwise F_ST_ were calculated using the diveRsity R package. Confidence intervals for A_R_, F_IS_, and F_ST_ were estimated using 1,000 bootstrap iterations using diveRsity. For H_O_ and H_E_, CIs were estimated with boot R package v. 1.3–32 (Canty & Ripley, 2025; Davison & Hinkley, 1997). A DAPC was performed with the adegenet R package as described above. The same analyses were performed with the nextRAD_complete dataset, subsampled for the same samples to allow direct comparison to the GT-seq panel. HAM6 was excluded from those calculations but was used to compare sex-assignment accuracy between the GT-seq panel and previous results based on Sanger sequencing (see Caeiro-Dias et al., 2023b for sex marker validation).

## Results

### Draft genome assembly

Whole genome sequencing originated a total of 208,986,054 barcoded paired-end reads, containing approximatly 60.3 Gb of data (~36x genome coverage for an expected genome size of 1.2 Gb). The assembly resulted in 578,094 contigs with a total length of about 0.86 Gb with a mean contig length of 1,492 bp and a N50 of 2,704 bp. The assembly also included a 16,713 bp long contig corresponding to the complete mitogenome reported by Osborne et al. (2020).

### Microhaplotype identification and primer design

Read alignment to the draft genome yielded on average 1.27 million (M) reads per individual (range = 0.9 M–5.3 M). A total of 1.2 M variants were discovered across the 379 individuals screened, from which 2,983 loci comprising 5,317 SNPs and 373 individuals (nextRAD_complete) passed all filtering steps (Table S1). Sample size and missing data for each year in the nextRAD_complete dataset are shown in Table 1. These loci were used for GT-seq panel development from which 2,664 loci passed filtering for SNP position on the nextRAD locus sequence and from those, primers were successfully design for 1,394 loci. From these primers, 719 were discarded because at least one of the primers mapped at multiple genomic regions. The remaining 675 loci were used to select a set of 500 loci for GT-seq panel optimization.

### Microhaplotype selection for GT-seq

Summary statistics of genetic diversity per locus were estimated from five subsets of 500 loci with primers and compared to the nextRAD_complete dataset to identify a set of markers that tracked temporal changes in genetic diversity. Regardless of the subset, the distribution of values for each metric were essentially the same across all temporal collections (Fig. 1, Table S3). Likewise, when compared to the nextRAD_complete dataset, the distributions of genetic diversity values across time-series were similar to the complete dataset albeit smaller (Fig. 1, Table S3). This was particularly true for A_R_.

**Figure 2:**
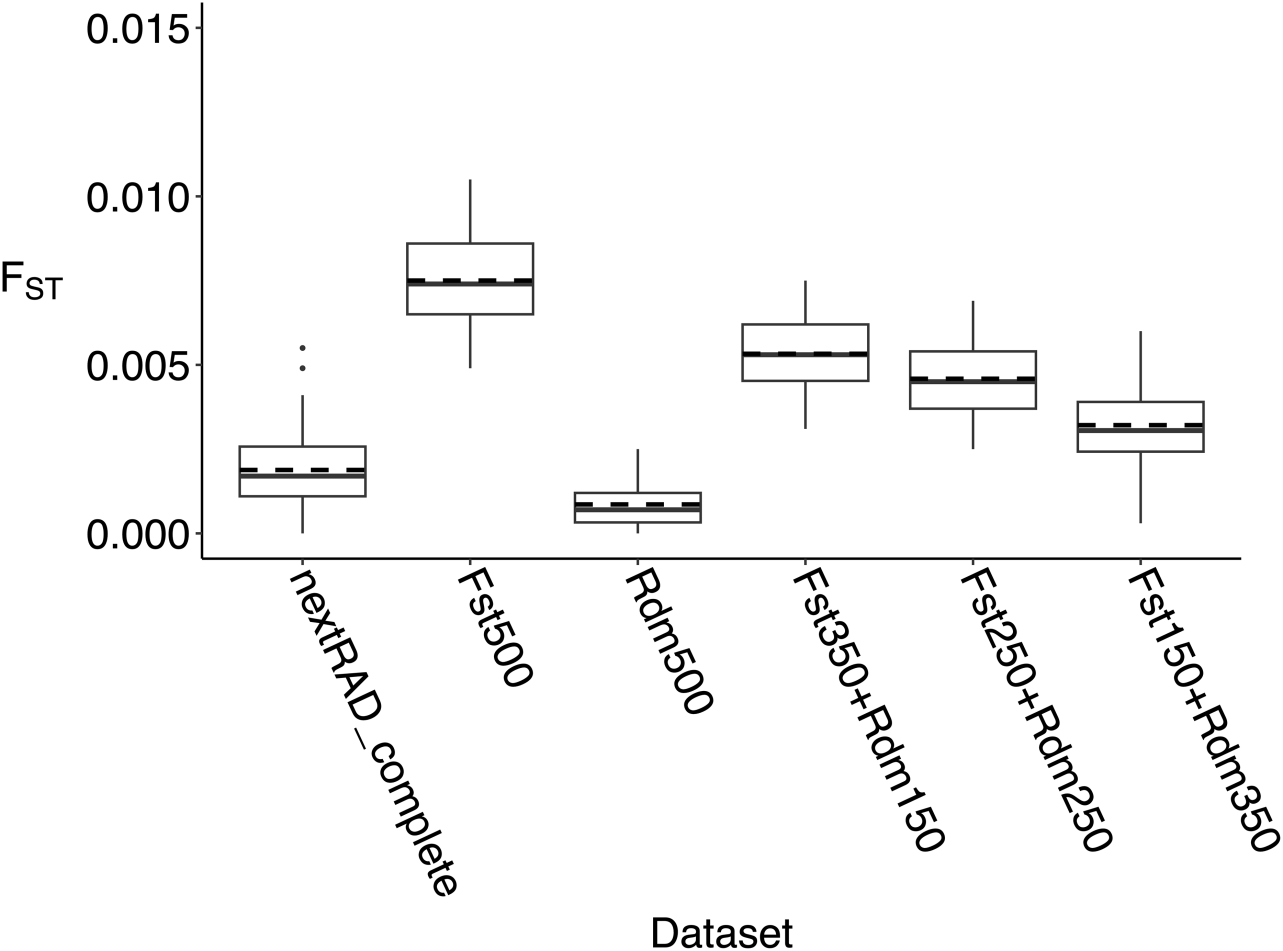
Population-level pairwise F_ST_ between all years (*i.e*., temporal pairwise F_ST_) estimated with the five subsets of 500 loci used to compare with results from nextRAD_complete dataset to identify a set of markers that tracked temporal changes in genetic diversity. Boxplots summarizing temporal pairwise F_ST_ estimates obtained with the the nextRAD_complete dataset (2,983 loci) and the five subsets of 500 loci (higher F_ST_ (F_ST_500), randomly selected loci (Rdm500), and different combinations of “F_ST_” plus “Random” (F_ST_350+Rdm150, F_ST_250+Rdm250, F_ST_150+Rdm350)). Solid line represents the median and the dashed line the mean. All F_ST_ values are provided on Table S4.

**Figure 3:**
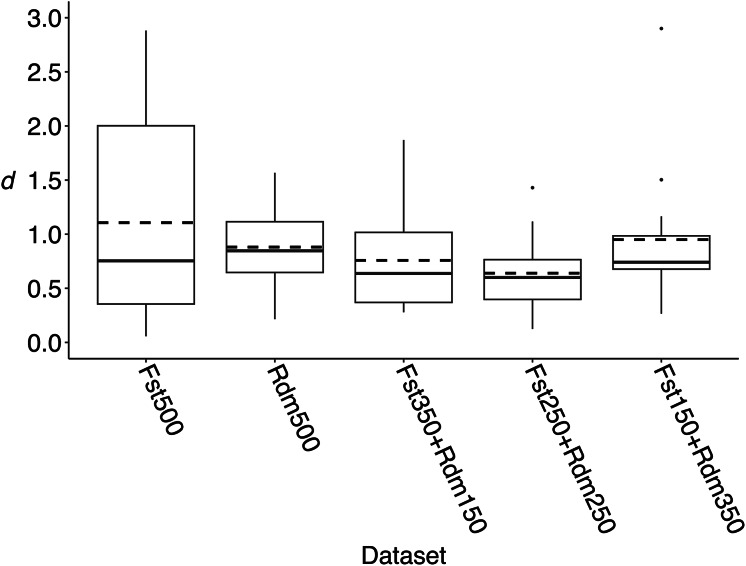
Euclidian distances (*d*) between DAPC group coordinates obtained from nextRAD_complete and each of the five subsets of 500 loci used to compare with results from nextRAD_complete dataset to identify a set of markers that tracked temporal changes in genetic diversity. Euclidian distances were estimated between nextRAD_complete (2,983 loci) DAPC group coordinates and the coordinates obtained for the corresponding year with each of the 500 loci subsets (loci with higher F_ST_ (F_ST_500), randomly selected loci (Rdm500), and different combinations of “F_ST_” plus “Random” (F_ST_350+Rdm150, F_ST_250+Rdm250, F_ST_150+Rdm350)). In each boxplot, the solid line is the median and the dashed line is the mean.

Pairwise F_ST_ estimates between all years were relatively small across datasets, but some differences were detected (Fig. 2 and Table S4). The F_ST_500 dataset had the greatest differences compared to the nextRAD_complete dataset but on average, F_ST_ values differed by only 0.007. For the other subsets, F_ST_ values were dependent on the random set of markers retained, but the differences to the nextRAD_complete dataset were even lower (Fig. 2). Unlike genetic diversity summary statistics and F_ST_ estimations, the DAPCs showed substantially different results across datasets (Fig. 3, Fig. S1). When comparing the Euclidian distances between DAPC group coordinates estimated with subsets and nextRAD_complete, the subset F_ST_250+Rdm250 showed smaller average *d* (0.64, ranging from 0.19 to 1.43), while F_ST_500 produced the highest (average *d* = 1.11, ranging from 0.31 to 2.88; Fig. 3). Although all datasets returned similar values of key metrics, DAPC indicated that the F_ST_250+Rdm250 subset best tracked temporal genetic changes across the 20-year time series. For this reason, this subset was selected for GT-seq optimization.

### GT-seq library preparation and PCR multiplex optimization

The first optimization round revealed that the multiplexed PCR performed poorly. The pool of demultiplexed reads across all individuals were dominated by primer interactions, which constituted 99.1% of demultiplexed reads (Table 2). From the initial 500 loci, 231 loci contributed to 99.0% of the primer interactions. From those, 151 (contributing to 97.9% of the total primer interactions) were removed due to disproportionate contribution to primer interactions with a single or multiple other primers. Because the library was overwhelmingly dominated by primer interactions, the average loci depth of coverage was 1.2 and average genotyping rate per locus was only 0.2%, it was not possible to estimate genotype accuracy. The second multiplex PCR improved substantially after the 151 primer pairs were removed. The proportion of primer interactions decreased to 46.2% and the average genotype accuracy was 82.1%. However, 38 loci contributed to most primer interactions (95.3% of the total interactions) in the second optimization round and these were discarded prior to preparation of the third GT-seq library. This further improved the multiplex by reducing the proportion of primer interactions to 35.5% and improving average per-locus genotype accuracy to 95.3%. The third optimization round identified additional 26 primer pairs contributing to primer interactions, over- and under-amplification or genotyping accuracy below 90% (see details in Table 2). The last round of optimization containing 284 loci confirmed that the proportion of primer interactions among the demultiplexed reads was relatively small (23.7%) and that the individual SNP genotype accuracy when compared to genotypes obtained from nextRAD was high (98.3%). Thus, the optimized GT-seq panel included 283 putatively neutral loci containing 352 SNPs and the sex-linked HAM6 locus containing two SNPs. From the neutral loci, 61 contained two to four SNPs (microhaplotypes) and 222 were single SNP loci. Hereafter, the optimized GT-seq panel containing the neutral loci is refered to as GT-seq_283. Locus-specific primer sequences for the optimized PCR multiplex and adapter sequences are provided as a text file in Data S1; the SNP-specific probes are provided as a text file with the format required for *AmpliconRadCounter.pl* script in Data S2.

**Table 2:** Summary results from GT-seq panel optimization. For each optimization round, the PCR multiplex performance was summarized with the overall proportion of reads resulting from primer interactions identified by *GTseq_Primer-Interactions.pl* script (primer interactions), and the average SNP genotyping accuracy across individuals used in nextRAD-seq and for the GT-seq optimization with less than 30% missing data (genotype accuracy). After the second optimization round, loci over- and under-amplifying and SNPs with genotype accuracy below 0.9 were discarded. Primers for a sex-linked marker (HAM6, Caeiro-Dias et al., 2023a) were included in the third and fourth rounds of optimization.

Optimization round		1^st^	2^nd^	3^rd^	4^th^
PCR multiplex performance	Primer interactions	99.10%	46.20%	35.50%	23.70%
	Genotype accuracy	NA^a^	82.10%	95.30%	98.30%
No. of primer pairs (loci) excluded	Primer interactions	154	38	8	0
	Over-amplification	–	–	4	0
	Under-amplification	–	–	5	0
	Genotype accuracy <0.9	–	–	9	0
	Total	154	38	26	0
No. of retained primer pairs (loci)		346	311	283 (+ HAM6)	283 (+ HAM6)

**Note:**

a Genotype accuracy could not be estimated because genotyping rate was low (average genotyping rate was 0.2%) due to low depth of coverage (average depth across all loci was 1.2).

### GT-seq panel validation

**Figure 4:**
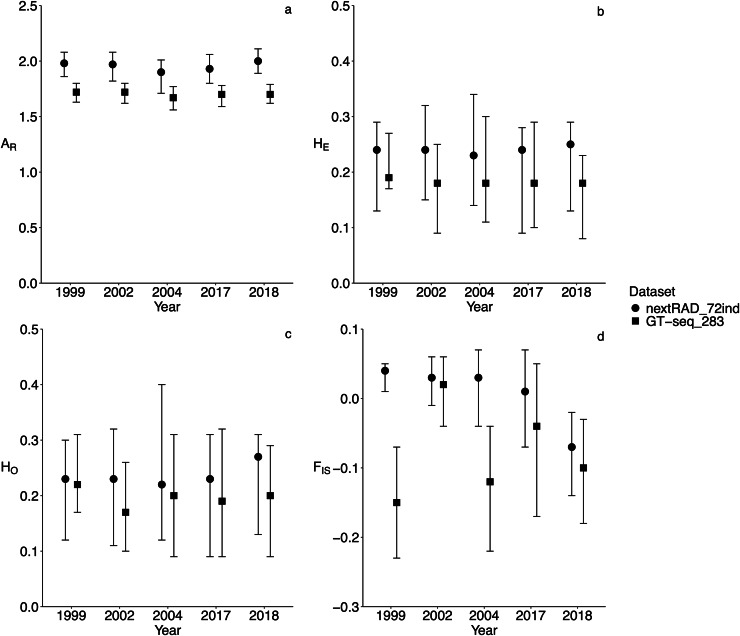
Concordance of genetic diversity metrics estimated with the genome-wide data (2,983 loci; nextRAD_72ind) and the optimized GT-seq panel (283 loci excluding the sex-linked marker; GT-seq_283). Diversity metrics were estimated from the 72 samples common to both datasets spanning five temporal collections. For details on sample sizes see Table 1. (A) A llelic richness (A_R_), (B) expected heterozygosity (H_E_), (C) observed heterozygosity (H_O_), and (D) inbreeding coefficient (F_IS_).

**Figure 5:**
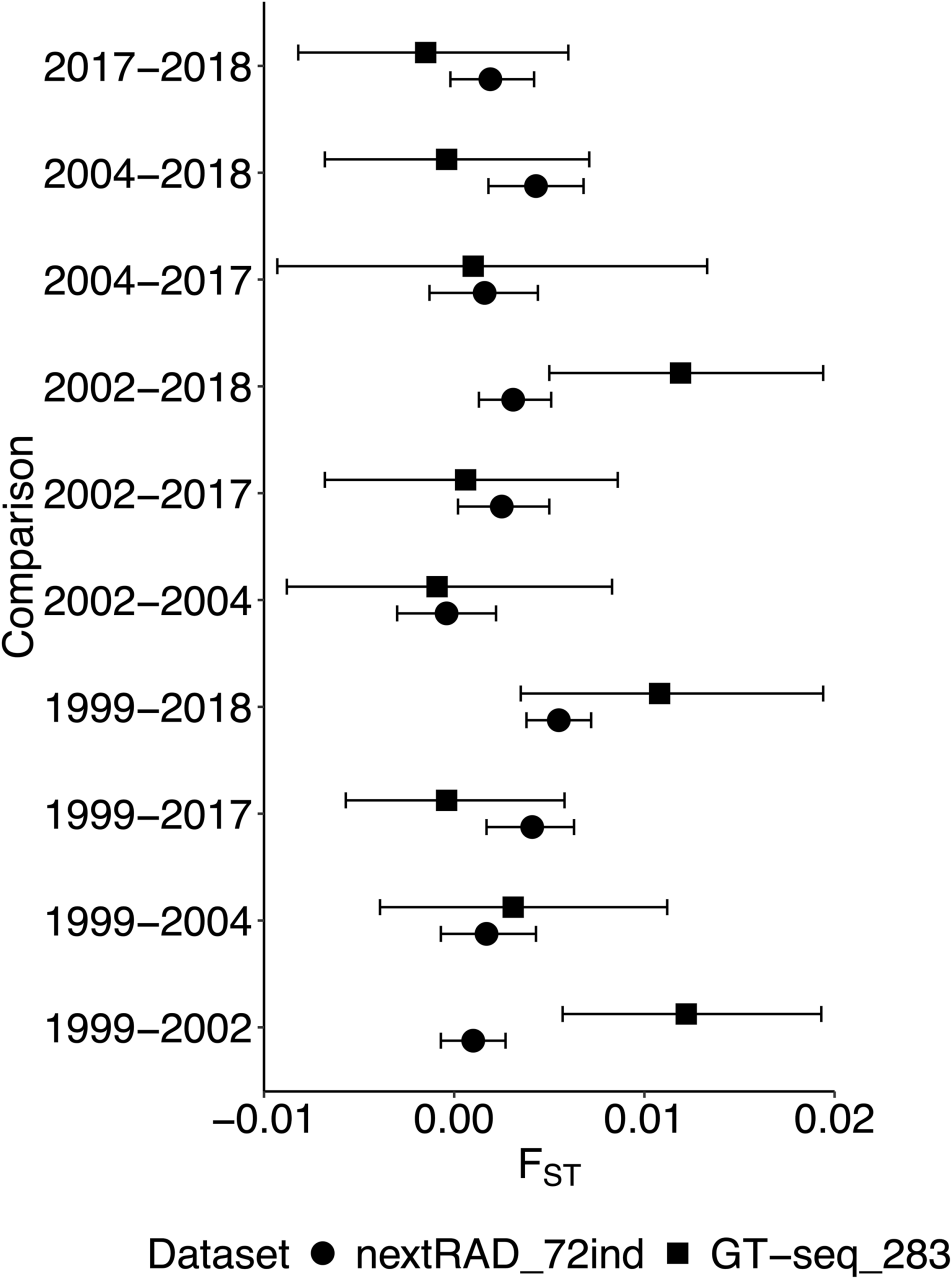
Pairwise F_ST_ estimates concordance between genome-wide data (2,983loci; nextRAD_72ind) and the optimized GT-seq panel (283 loci excluding the sex-linked marker; GT-seq_283). Pairwise F_ST_ values were estimated between all five temporal collections represented by the 72 samples genotyped with GT-seq panel and sub-sampled from nextRAD_complete. Error bars represent the 95% confidence intervals.

**Figure 6:**
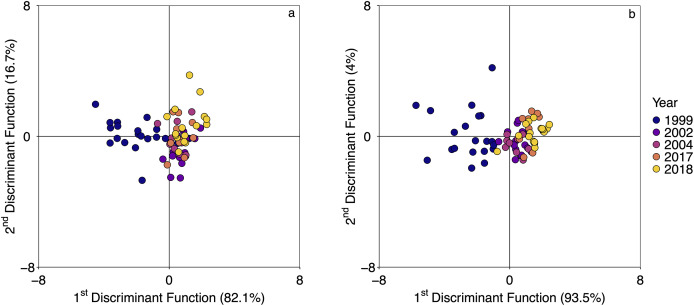
Temporal structure concordance between genome-wide data (2,983 loci; nextRAD_72ind) and the optimized GT-seq panel (283 loci excluding the sex-linked marker; GT-seq_283). Discriminant analysis of principal components (DAPC) results obtained from (A) the optimized GT-seq panel and (B) DAPC results from obtained from genome-wide data including the same 72 individuals used for panel validation. The percentages on the x-axis and y-axis refer to the proportion of variation explained by the first and second discriminant functions, respectively.

From the 133 samples genotyped with the GT-seq panel, 23 samples from the temporal dataset were removed due to missing data higher than 30% and 38 were not included in the analysis because the corresponding temporal collection included less than 10 individuals. Five temporal collections (1999, 2002, 2004, 2017, and 2018) with 10 or more individuals (total of 72 individuals retained; nextRAD_72ind dataset; Table 1) were used for the DAPC and to estimate genetic diversity summary statistics with the GT-seq_283 and the nextRAD_72ind. Missing data was consistently low across datasets from 2002–2018 collections (0.01–0.02 in nextRAD_72ind and 0.003-0.03 in GT-seq_283). In 1999, missing data was the highest in both cases (0.05 in nextRAD_72ind and 0.1 in GT-seq_283). Both datasets returned similar estimates of genetic diversity across the 5 years (Fig. 4). As expected from the results of loci selection, A_R_ estimated with the GT-seq panel was smaller than A_R_ estimated with genome-wide data and 95% CIs obtained from both datasets often did not overlap (Fig. 4A). For H_E_ and H_O_ results were very similar (although values were slightly lower in the GT-seq panel) and CIs broadly overlaped (Figs. 4B and 4C). For most years, F_IS_ estimated with both datasets were similar and 95% CIs also broadly overlapped (Fig. 4D). In two instances, F_IS_ values were considerably smaller. In one case (1999) 95% CIs did not overlap and in another case (2004) the upper bound of one CI was the same as the lower of the other CI. Also, pairwise F_ST_ estimates obtained with both datasets were comparable with broad 95% CIs overlap (Fig. 5). The only two exceptions were 1999–2002 and 2002–2018. In the first case 95% CIs did not overlap and in the second case, the upper bound of one CI was the same as the lower of the other CI. Broad patterns of temporal structure identified with DAPC (Fig. 6) were also similar between GT-seq_283 and the nextRAD_72ind. Both datasets identified a gradual shift in genetic variation from 1999 to 2018. The main difference was that with nextRAD complete the shift was captured by the x-axis while with the GT-seq_283 the shift from 2002 to 2018 was mostly captured in the y-axis. This was reflected by more variation explained by the y-axis when using the GT-seq_283 data. Nonetheless, both DAPC results show that the biggest changes happened from 1999 to 2002 while from 2002 to 2018 there is a broader overlap among years. Genotype accuracy for HAM6 using the GT-seq panel when compared to Sanger-seq results from Caeiro-Dias et al. (2023a) was consistent across all 15 samples (Table S5). An alignment with forward and reverse sequences (the reverse sequences are provided as reverse-complement) from each individual obtained with Sanger-seq for this locus is provided as a fasta file in Data S3, including the reference sequence from Caeiro-Dias et al. (2023b). Position 72 in the alignment corresponds to the SNP A/C and position 119 corresponds to SNP G/T from Caeiro-Dias et al. (2023b).

## Discussion

Rio Grande silvery minnow genetic monitoring was envisioned and designed to identify significant changes in genetic diversity over time. The most important goal in this study was to design a GT-seq panel that was both sensitive to temporal genetic change, and consistent with trends revealed by microsatellites (Osborne et al., 2024) and genome-wide microhaplotypes (Osborne, Caeiro-Dias & Turner, 2023). Consistency between microsatellites and microhaplotypes was evaluated by Osborne, Caeiro-Dias & Turner (2023). Here, a GT-seq panel was developed to replace the existing microsatellite-based genetic monitoring protocol for Rio Grande silvery minnow and the consistency with genome-wide microhaplotype data was evaluated. Temporal data from archived collections spanning 20 years of genetic monitoring (1999–2018) was used to design and optimize a panel of 283 loci, including microhaplotypes and single SNPs, that successfully tracked genetic diversity metrics and temporal variation. Inclusion of archived samples provided critical information about the reliability of the panel to recover key changes in genetic diversity over time. Loci included in the optimized GT-seq panel were consistent with those obtained from nextRAD-seq with low genotype discordance (1.7%). These results are comparable with other studies developing GT-seq panels from reduced representation sequencing methods (Hayward et al., 2022; Schmidt et al., 2020; Setzke, Wong & Russello, 2021). Estimates of H_E_ and H_O_ obtained with GT-seq data were virtually the same as those obtained from genome-wide microhaplotype data (2,983 loci comprising 5,315 SNPs). On the other hand, A_R_ was consistently lower with GT-seq data. This result is not unexpected because fewer loci were assayed using the panel. In some cases, fewer SNPs were retained the in GT-seq_283 compared to the same locus in the nextRAD_72ind data due to SNP filtering for primer design. Most F_IS_ estimates were congruent between datasets, except for 1999 and 2004. Samples from 1999 genotyped with the GT-seq panel had the highest proportion of missing data, yielding a strong positive correlation between missing data and heterozygosity (Pearson’s R = 0.22, *p*-value = 1.96 × 10^−4^). Moreover, we found that loci with lower coverage exhibited the most missing data and showed a general increase of heterozygosity (Fig. S2). Low depth of coverage in reduced-representation sequencing is known to result in increased SNP genotyping errors (Beissinger et al., 2013; Bresadola et al., 2020). The results observed for the 1999 sample likely resulted from geotyping errors inflating false heterozygotes which can explain lower F_IS_ values obtained with GT-seq panel. For 2004, lower F_IS_ obtained with the GT-seq panel is probably related with a low sample size. In this case, very low sample sizes likely resulted in failure to capture low-frequency alleles and the highest observed proportion of monomorphic loci (29.3%) when compared to all other collections (range = 14.8–23.6%). Because monomorphic loci are not accounted for in F_IS_ estimations, loci with higher heterozygosity are likely overrepresented in 2004, resulting in a lower F_IS_ value. Indeed, data from 91 individuals genotyped with the GT-seq panel (F_IS_ = 0.011, 95% CI [−0.0138 to 0.0323]) by Osborne, Caeiro-Dias & Turner (2025) showed a much smaller difference from the 26 individuals genotyped in this study for the 2,983 loci in the nextRAD dataset with overlapping 95% CIs (F_IS_ = 0.051 [0.031–0.067]).

Population-level pairwise F_ST_ estimates were consistent across datasets, as most point values were similar and 95% CIs overlapped. Two exceptions to this pattern were identified (1999–2002 and 2002–2018) but it was difficult to pinpoint the cause of discrepancies. It is likely that small sample size for some collections could explain the bias. When larger sample size was screened with the GT-seq panel (1999, *N* = 33; 2002, *N* = 101; 2018, *N* = 105) by Osborne, Caeiro-Dias & Turner (2025), pairwise F_ST_ values were identical to those obtained from all samples in the nextRAD data presented in this study (see Table 1). For 1999–2002 F_ST_ (GT-seq panel) = 0.0027 (95% CI [−0.0026 to 0.0091]) and F_ST_ (nextRAD) = 0.001 (95% CI [0.0009–0.002]). For 2002–2018 F_ST_ (GT-seq panel) = 0.004 (95% CI [0.0019–0.0065]) and F_ST_ (nextRAD) = 0.0017 (95% CI [0.0013–0.0021]). In both cases F_ST_ differences are smaller than presented in Fig. 5, with overlapping 95% CIs.

Broad patterns of temporal variation detected with the nextRAD_72ind, were also recovered with the GT-seq_283. With both datasets, the main difference detected was between 1999 and everything else. A gradual shift from 2002 to 2018 was detected with both datasets. While with the nextRAD_72ind the shift was shown mostly along the x-axis, with the GT-seq_283 that was more obvious along the y-axis. These results show that although the 2,983 loci have more power to detect smaller differences, the power of the GT-seq panel is still sufficient to detect the same temporal variation. Moreover, results from the application of the GT-seq panel for genetic monitoring (Osborne, Caeiro-Dias & Turner, 2025) are similar to results obtained nextRAD data (Osborne, Caeiro-Dias & Turner, 2023) and microsatellites (Osborne, Caeiro-Dias & Turner, 2023; Osborne et al., 2024). A sex-linked marker (HAM6) was also included in the panel which allowed accurate identification of an individual’s sex (for sex-marker identification and validation see Caeiro-Dias et al., 2023b).

In endangered species like Rio Grande silvery minnow, rapid and substantial geographic range contractions and population declines are frequently followed by a loss of genetic diversity (Casas-Marce et al., 2013; Lobo, López-Bao & Godinho, 2023). Reduced diversity limits the number of variable loci discoverable by reduced-representation sequencing methods like nextRAD-seq. Stringent filtering depends on research goals, but it is generally a good practice when identifying SNPs (O’Leary et al., 2018). Yet, strict filtering likely results in fewer loci available to develop a GT-seq panel in species with low genetic diversity. Moreover, not all loci identified are compatible with the GT-seq library preparation (Bootsma et al., 2020). Loci may also be excluded when designed primers amplify multiple loci. Hence, identifying more candidate loci in initial steps may improve chances of developing a GT-seq panel with adequate discriminatory power to meet monitoring goals. Initially, the aim of this study was to maximize the number of candidate loci for the GT-seq panel by using a conspecific reference genome to facilitate locus assembly. A conspecific reference genome-guided variant calling yields more SNPs (Shafer et al., 2017). Furthermore, the number of loci available was substantially increased by filtering SNPs based on locus position. This strategy enabled us to find flanking regions for primer design while retaining other candidate SNPs present in that locus. With a few exceptions (*e.g*., Schmidt et al., 2020) SNP filtering based on locus position is normally not considered when identifying loci for GT-seq panels and loci with insufficient flanking sequence for primer design are usually discarded (Bootsma et al., 2020; Chang, Ward & Russello, 2021).

A conspecific reference genome is also useful for mapping designed primers to test for locus specificity. Identification of locus-specific primers before optimization reduces the optimization effort and costs by avoiding ordering primers early in the process that would otherwise be discarded. From the initial 2,983 candidate loci from nextRAD-seq, primers were successfully designed for 46.7% of those, and only 22.6% were retained after excluding primers mapping to multiple genomic regions.

A short-read whole genome sequencing approach was shown to be sufficient for locus identification. When developing a GT-seq panel for species/populations with reduced genomic diversity from reduced representation sequencing methods to identify variable loci, using a conspecific reference genome is advantageous. Specifically, a conspecific reference genome (i) maximizes the number of initial loci identified, as a large proportion of the loci obtained from SNP discovery might not be compatible with GT-seq library preparation methods, and (2) allows identification of primers that potentially anneal to multiple genomic regions. More complete genomes than the one used here will also be advantageous to ensure that loci are not in physical proximity on chromosomes (*i.e*., linked). Yet, depending on the biological system, *de novo* SNP identification can yield enough loci to develop a suitable GT-seq panel, particularly if *de novo* reference assemblies are generated from high quality samples (Schmidt et al., 2020).

In the case of Rio Grande silvery minnow, summary statistics obtained from the tested subsets of 500 loci were found to be less sensitive to marker selection than the magnitude of temporal genetic changes detected from F_ST_ estimates and DAPC. Irrespective of the subset tested, estimations of genetic diversity were strikingly similar across subsets. Values of F_ST_ exhibited some variation between datasets, and temporal genetic changes across the 20-year time-series assessed with the DAPC were more faithfully tracked by F_ST_250+Rdm250 subset. The aim of the GT-seq panel developed here was not to identify genetic stocks or geographical population structure; a common goal for many other GT-seq panels that have been developed (Beemelmanns et al., 2024; Hayward et al., 2022; McKinney et al., 2022; Schmidt et al., 2020). Rather, our goal was to track known shifts in genomic variability across time to preserve the continuity of genetic monitoring. As such, searching for a panel of markers that provided a signature of genomic shifts comparable to that obtained with the genome-wide SNP-based data (Osborne, Caeiro-Dias & Turner, 2023) as well as microsatellites (Osborne et al., 2024) was essential. For this reason, including a set of loci with higher F_ST_ across time was important to achieve this goal. However, including a set of random loci was equally important to provide a similar pattern of temporal genomic changes equivalent to that provided by genome-wide data.

## Conclusions

Overall, using archived samples in panel development offered an opportunity to capitalize on information from a long time series to maximize sensitivity to temporal genetic change. Also selecting a set of loci with higher F_ST_ allowed tracking known temporal changes in genetic variability while including of a set of random loci was equally important to avoid bias in the signal of temporal changes. Regardless of the loci selection scheme, the diversity metrics were accurate relative to a genome-wide dataset. After initial SNP discovery and panel optimization, the final GT-seq panel provided an effective approach for detection of temporal alleleic frequency shifts and accurate estimation of metrics commonly used in population genomic studies. A GT-seq approach combined with the analysis of archived collections can thus be a powerful tool facilitating continuity of genetic monitoring to inform conservation efforts. Although the molecular methods used here are standard for developing a GT-seq panel, this is the first study (to our knowledge) using archived samples in development of a GT-seq panel that maintains the analytical and inferential continuity of long-term genetic monitoring.

The GT-seq panel developed here for the endangered Rio Grande silvery minnow offers a low-cost and efficient genotyping tool for genetic monitoring of the wild and hatchery populations. The panel allows timely documentation of changes to genetic diversity resulting from fluctuating abundance, other demographic processes and augmentation of the wild population with hatchery-reared fish. Hence, the development of the GT-seq panel will faciliate adaptive management and targeted conservation actions that aim to protect standing genetic diversity and to avoid further losses.

## Supplemental Information

10.7717/peerj.20726/supp-1 Supplemental Information 1 Details for microhaplotype identification from nextRAD; and microhaplotype selection for GT-seq and PCR multiplex optimization.

10.7717/peerj.20726/supp-2 Supplemental Information 2 GT-seq primers for *Hybognathus amarus*.

10.7717/peerj.20726/supp-3 Supplemental Information 3 Tab delimited table containing the eight columns in the same format required by *AmpliconReadCounter.pl* script (GTscore pipeline v. 1.3; https://github.com/gjmckinney/GTscore).

10.7717/peerj.20726/supp-4 Supplemental Information 4 Sex-linked marker (HAM6) sequences (forward and reverse) obtained from Sanger sequencing (Caeiro-Dias et al. 2023) for 15 individuals genotyped with the optimized GT-seq panel.

## Additional Information and Declarations

### DNA Deposition

The following information was supplied regarding the deposition of DNA sequences:

Raw sequence reads from GT-seq used for panel validation (117 individuals) and sex assignment consistency (15 individuals), are deposited in the NCBI Sequence Read Archive (SRA), with the BioProject accession number PRJNA887477. BioSample accession numbers can be found in Supplemental Information Table S2 and Table S5.

Contig-level assembly from whole-genome short-read sequencing (BioSample accession number SAMN45830324) is deposited in SRA with the BioProject accession number PRJNA887477.

### Data Availability

The following information was supplied regarding data availability:

The individual genotype datasets are available on Dryad: Caeiro-Dias, Guilherme; Osborne, Megan; Turner, Thomas (2025). Data from: Time is of the essence: using archived samples to develop a GT-seq panel to preserve continuity of ongoing genetic monitoring [Dataset]. Dryad. https://doi.org/10.5061/dryad.4mw6m90mv.

The code is available at GitHub and Zenodo:

- https://github.com/gcaeirodias/multi_HWE_tests.

- Guilherme Caeiro-Dias. (2025). gcaeirodias/multi_HWE_tests: v.1.0.0—First release (v.1.0.0). Zenodo. https://doi.org/10.5281/zenodo.17914374.

- https://github.com/gcaeirodias/significantLD.

- Guilherme Caeiro-Dias. (2025). gcaeirodias/significantLD: v.1.0.0—First release (v.1.0.0). Zenodo. https://doi.org/10.5281/zenodo.17915458.

- https://github.com/gcaeirodias/GT-seq_filters.

- Guilherme Caeiro-Dias. (2025). gcaeirodias/GT-seq_filters: v.1.0.0—First release (1.0.0). Zenodo. https://doi.org/10.5281/zenodo.17943592.

The voucher used for genome-sequencing is available at the Museum of Southwestern Biology (catalog number MSB:Fish 114645).
