# Supplementary material for: Time is of the essence: using archived samples in the development of a GT-seq panel to preserve continuity of ongoing genetic monitoring
Source: PeerJ. 2026 Feb 4;14:e20726. doi: 10.7717/peerj.20726 (PMC12882729; doi:10.7717/peerj.20726)
Supplement: Supplemental Information 1 [file peerj-14-20726-s001.pdf]

Supplemental Information for:

Time is of the essence: using archived samples to develop a GT-seq panel to maintain continuity of an ongoing genetic monitoring

Guilherme Caeiro-Dias[1*], Megan J. Osborne[1], and Thomas F. Turner[1]

[1]Department of Biology and Museum of Southwestern Biology, MSC 03-2020, University of New Mexico, Albuquerque, New Mexico, 87131, USA.

*Corresponding author; Email: gcaeirodias@unm.edu

Table of Contents:

Microhaplotype identification from nextRAD-seq	Page 2
Table S1	Page 2
Microhaplotype selection for GT-seq and PCR multiplex optimization	Page 5
Table S2	Page 5
Table S3	Page 6
Table S4	Page 8
Figure S1	Page 11
Table S5	Page 12
Figure S2	Page 12
References	Page 12

Microhaplotype identification from nextRAD-seq

Table S1 – Description of the pipeline used to obtain the dataset from which loci compatible with the GT-seq protocol were identified. The first column contains the general step, and the second column describes the objective of that step or filter applied. The third column contains the command used in each step and the reference to the bioinformatic tool/software (references are provided in the footnote). The corresponding options and thresholds applied are provided in the fourth column, and when not otherwise specified, default options/values were applied. The last three columns contain the resulting number of reads, variants, and individuals, respectively, obtained after each filter.

Step	Filter/goal description	Command	Options and thresholds	Nr of reads	Nr of variants	Nr of individuals
Demultiplex	Assign nextRAD raw reads to corresponding individuals and sequencing lanes.	Received already demultiplex from SNPsaurus (sequencing provider).		1.34 billion	-	379
Trim	Remove low quality bases and trim reads based on average quality on both ends.	java -Xmx16g -jar trimmomatic-0.39.jar[1]	SE<br>LEADING:20<br>TRAILING:20<br>SLIDINGWINDOW:5:10<br>MINLEN:60	1.34 billion	-	379
Align to reference	Align reads to *H. amarus* draft reference genome.	bowtie2[2]	-q<br>--phred33<br>--local<br>--very-sensitive<br>--no-1mm-upfront<br>-x [fasta file genome]	1.27 billion	-	379
		samtools[3]	view<br>-q 20			
Prepare files for variant call	Add read group flags to BAM files, sort, obtain one BAM per individual* and one BAM containing alignments from all individuals and use this complete BAM to create a BED file with nextRAD loci to call SNPs.	java -Xmx16g -jar $PICARD[4]	AddOrReplaceReadGroups<br>RGLB=[library]<br>RGPL=[platform]<br>RGPU=[instrument]<br>RGSM=[sample name]	1.27 billion	-	379
		java -Xmx16g -jar $PICARD[4]	SortSam<br>SORT_ORDER=coordinate			
		java -Xmx16g -jar $PICARD[4]	MergeSamFiles*<br>$(echo ${[array with bam names to be merged][@]})<br>ASSUME_SORTED=true<br>USE_THREADING=true			
		bedtools[5]	merge			

Table S1 – Continuation.

Step	Filter/goal description	Command	Options and thresholds	Nr of reads	Nr of variants	Nr of individuals
Variant call	Identify variants from nextRAD loci.	freebayes[6] (using the code to parallelize run implemented on dDocent v. 2.7.8[7])	--min-mapping-quality 5<br>--min-base-quality 5	-	1.2 million	379
Variant and individual filtering	Remove variants with low quality.	vcftools[8]	--minGQ 20<br>--minDP 5<br>--mac 3<br>--min-meanDP 20<br>--max-minDP 200<br>--maf 0.02	-	11,143	379
	Remove individuals with high missing data (MD).	vcftools[8]<br>vcftools[8]	--missing-indv<br>--remove [file with individuals > 25% MD]	-	11,143	373
	Decompose multi-nucleotide states into SNPs.	vcfallelicprimitives[9]	-k<br>-g	-	12,564	373
	Keep biallelic SNPs and discard loci with high missing data.	vcftools[8]	--remove-indels<br>--min-alleles 2<br>--max-alleles 2<br>--max-missing 0.9	-	11,361	373
	Filter out potential erroneous SNPs based on allelic balance at heterozygous genotypes, strand representation, quality vs depth and site depth. (see authors recommendations and details about each filter[10]).	dDocent_filters[10†] (depends on vcflib[9], vcftools[8])	-	-	8,609	373
	Filter out potential erroneous SNPs based on Hardy-Weinberg equilibrium (see authors recommendations and details about this filter[1]).	filter_hwe_by_pop.pl[11]	-p [file with population of each individual]<br>-h 0.001	-	8,566	373

Table S1 – Continuation.

Step	Filter/goal description	Command	Options and thresholds	Nr of reads	Nr of variants	Nr of individuals
Variant and individual filtering	Remove putative paralogous loci and exclude loci exceeding the expected number of haplotypes.	rad_haplotyper.pl[12]	-h 10<br>-mp 5	-	5,350	373
		vcftools[8]	--bed [bed with intervals of loci identified by rad_haplotyper.pl]			
	Haplotyping SNPs within a locus	rad_haplotyper.pl[12]	-mp 5	-	2,992 micro-haplotypes (5,329 SNPs)	373
	χ2 test on microhaplo-type data for deviations from Hardy-Weinberg equilibrium.	multi_HWE_tests.sh[13] (depends on R packages adegenet[14,15], pegas[16], dplyr[17])	-	-	2,992 micro-haplotypes (5,329 SNPs)	373
	χ2 tests on SNP data (using the SNP of each locus with higher MAF) for linkage disequilibrium	makeUR[18]	MAF=0.02<br>MAXDEPTH=200	-	2,983 micro-haplotypes (5,317 SNPs)	373
		GUSLD[19] significantLD.sh[20] (depends on R packages dplyr[17], stringr[21], data.table[22], rcompanion[23])	-			

[1]Trimmomatic v. 0.36 (Bolger et al., 2014); [2]Bowtie v. 2.3.1 (Langmead & Salzberg, 2012); [3]Samtools v. 1.10 (Li et al., 2009); [4]Picard v. 2.20.8 (https://broadinstitute.github.io/picard/); [5]Bedtools (Quinlan & Hall, 2010); [6]FreeBayes v. 1.1.0 (Garrison & Marth, 2012); [7]dDocent v. 2.7.8 (Puritz et al., 2014); [8]VCFtools v. 0.1.16 (Danecek et al., 2011); [9]vcflib (https://github.com/vcflib/vcflib); [10]dDocent_filters (https://github.com/jpuritz/dDocent/blob/master/scripts/dDocent_filters); [11]filter_hwe_by_pop.pl (https://github.com/jpuritz/dDocent/blob/master/scripts/filter_hwe_by_pop.pl); [12]rad_haplotyper.pl (Willis et al., 2017); [13]multi_HWE_tests.sh (https://github.com/gcaeirodias/multi_HWE_tests); [14,15]adegenet (Jombart, 2008; Jombart & Ahmed, 2011); [16]pegas v. 1.0 (Paradis, 2010); [17]dplyr v. 1.0.0 (Wickham et al. 2020); [18]GUSbase 0.2.0 (Bilton, 2019); [19]GUSLD v. 1.0.1 (Bilton et al., 2018); [20]significantLD (https://github.com/gcaeirodias/significantLD); [21]stringr v. 1.4.0 (Wickham, 2019); [22]data.table v. 1.13.0 (Barrett et al., 2020); [23]rcompanion v. 2.3.21 (Mangiafico 2020). All analysis for variant filtering using R were conducted in R studio v. 1.2.5033-1 (RStudio Team 2019) and the version 3.6.3 of that software (R Core Team 2019).

*Picard tools' MergeSamFiles command was used to merge bam files with data obtained from different sequencing lanes from each individual to obtain one bam file per sample; then it was used to merge all individual bam files to obtain a single bam file with data from all individuals.

Table S1 – Continuation.

†dDocent_filters script discards SNPs if: heterozygous SNPs had alternate allele with COV < 0.2 or > 0.8 compared to reference allele (alleles with frequencies < 0.01 and > 0.99 were not removed to account for fixed alleles); quality sum of the reference or alternate allele was zero; ratio between the mean MAPQ of the alternate and reference allele < 0.9 or > 1.05; loci with quality scores less than half of the total DP; DP > the average DP plus one standard deviation and if the quality score was less than 2x the DP; and mean depth across individuals greater than 2x the mode (101 for the dataset in this study; ~95th percentile of mean depth).

Microhaplotype selection for GT-seq and PCR multiplex optimization

Table S2 – NCBI Sequence Read Archive (SRA) BioSample accession numbers for the 87 samples used for optimization of the GT-seq panel. Samples were previously sequenced using a nextRAD protocol (BioProject PRJNA887477) reported in Osborne et al. (2022).

BioSample (SAMN)	Sample Name	BioSample (SAMN)	Sample Name	BioSample (SAMN)	Sample Name
31170431	2002_ANG_534	31170534	2008_ISL_07	31170675	2017_ISL_007
31170443	2002_ISL_453	31170535	2008_ISL_08	31170679	2017_SA_01
31170444	2002_ISL_454	31170538	2008_SA_03	31170680	2017_SA_02
31170445	2002_ISL_456	31170542	2008_SA_07	31170682	2017_SA_04
31170446	2002_ISL_461	31170736	2008_WRJ08703_01	31170683	2017_SA_05
31170448	2002_ISL_464	31170608	2012_ANG_206	31170685	2017_SA_07
31170449	2002_ISL_509	31170610	2012_ANG_209	31170686	2017_SA_08
31170450	2002_ISL_510	31170611	2012_ANG_210	31170690	2018_ANG_003
31170452	2002_SA_206	31170615	2012_ISL_067	31170691	2018_ANG_004
31170453	2002_SA_211	31170621	2012_ISL_185	31170692	2018_ANG_005
31170456	2002_SA_216	31170623	2012_ISL_187	31170693	2018_ANG_006
31170458	2002_SA_218	31170625	2012_SA_281	31170694	2018_ANG_007
31170459	2002_SA_219	31170626	2012_SA_290	31170696	2018_ANG_009
31170460	2002_SA_221	31170627	2012_SA_291	31170697	2018_ANG_010
31170462	2004_ANG_034	31170629	2012_SA_293	31170701	2018_ISL_003
31170464	2004_ANG_047	31170633	2015_ANG_045	31170702	2018_ISL_004
31170465	2004_ANG_049	31170638	2015_ANG_051	31170703	2018_ISL_005
31170466	2004_ANG_053	31170642	2015_ANG_110	31170704	2018_ISL_006
31170468	2004_ANG_055	31170643	2015_ANG_118	31170705	2018_ISL_007
31170475	2004_ISL_17	31170644	2015_ISL_002	31170708	2018_ISL_010
31170478	2004_ISL_40	31170649	2015_ISL_007	31170709	2018_SA_001
31170481	2004_ISL_43	31170732	2015_RKD1506_06	31170710	2018_SA_002
31170482	2004_ISL_44	31170733	2015_RKD1506_07	31170711	2018_SA_003
31170484	2004_SA_008	31170734	2015_RKD1506_09	31170712	2018_SA_004
31170485	2004_SA_009	31170735	2015_RKD1506_10	31170713	2018_SA_005
31170519	2008_ANG_02	31170663	2017_ANG_005	31170714	2018_SA_006
31170520	2008_ANG_03	31170665	2017_ANG_007	31170715	2018_SA_007
31170522	2008_ANG_05	31170671	2017_ISL_003	31170718	2018_SA_010
31170523	2008_ANG_06	31170672	2017_ISL_004	31170719	2018_SA_011

Table S3 – Average allelic richness (A_R), observed heterozygosity (H_O), expected heterozygosity (H_E), and inbreeding coefficient (F_{IS}) estimated for each temporal collection with the complete dataset (nextRAD_complete) and each of the datasets containing subsets of 500 loci. One subset had 500 loci with higher F_{ST} (F_{ST}500), and another had 500 randomly selected loci (Rdm500); the other three subsets were different combinations of "F_{ST}" plus "Random": F_{ST}350+Rdm150; F_{ST}250+Rdm250; and F_{ST}150+Rdm350.

Year	Dataset	Nr of microhaplotypes	A_R	H_O	H_E	F_{IS}
1999 (n=28)	Complete	2983	2.28	0.23	0.24	0.0415
	F_{ST}500	500	2.02	0.20	0.21	0.0334
	Rdm500	500	2.03	0.20	0.21	0.0315
	F_{ST}350+Rdm150	500	2.03	0.21	0.21	0.0276
	F_{ST}250+Rdm250	500	2.03	0.20	0.21	0.0446
	F_{ST}150+Rdm350	500	2.02	0.20	0.21	0.0444
2000 (n=42)	Complete	2983	2.37	0.25	0.25	0.0073
	F_{ST}500	500	2.10	0.22	0.22	-0.0085
	Rdm500	500	2.12	0.22	0.22	0.0012
	F_{ST}350+Rdm150	500	2.10	0.22	0.22	0.0000
	F_{ST}250+Rdm250	500	2.10	0.22	0.22	0.0013
	F_{ST}150+Rdm350	500	2.09	0.21	0.21	-0.0031
2002 (n=30)	Complete	2983	2.34	0.23	0.25	0.0552
	F_{ST}500	500	2.07	0.20	0.21	0.0472
	Rdm500	500	2.11	0.21	0.22	0.0497
	F_{ST}350+Rdm150	500	2.07	0.20	0.21	0.0641
	F_{ST}250+Rdm250	500	2.07	0.20	0.21	0.0478
	F_{ST}150+Rdm350	500	2.06	0.19	0.21	0.0575
2004 (n=26)	Complete	2983	2.31	0.23	0.24	0.0513
	F_{ST}500	500	2.01	0.20	0.2	0.0350
	Rdm500	500	2.06	0.20	0.21	0.0344
	F_{ST}350+Rdm150	500	2.02	0.20	0.21	0.0453
	F_{ST}250+Rdm250	500	2.02	0.20	0.20	0.0407
	F_{ST}150+Rdm350	500	2.02	0.20	0.20	0.0277
2006 (n=30)	Complete	2983	2.32	0.24	0.25	0.0167
	F_{ST}500	500	2.05	0.21	0.21	0.0033
	Rdm500	500	2.09	0.22	0.22	0.0075
	F_{ST}350+Rdm150	500	2.05	0.21	0.21	0.0002
	F_{ST}250+Rdm250	500	2.05	0.20	0.21	0.0218
	F_{ST}150+Rdm350	500	2.04	0.20	0.21	0.0193
2008 (n=30)	Complete	2983	2.34	0.25	0.25	0.0005
	F_{ST}500	500	2.07	0.22	0.22	0.0030
	Rdm500	500	2.10	0.23	0.23	0.0014
	F_{ST}350+Rdm150	500	2.08	0.22	0.22	0.0017
	F_{ST}250+Rdm250	500	2.08	0.21	0.22	0.0126
	F_{ST}150+Rdm350	500	2.07	0.21	0.21	-0.0024

Table S3 – Continuation.

Year	Dataset	Nr of microhaplotypes	A_R	H_O	H_E	F_{IS}
2009 (n=29)	Complete	2983	2.28	0.21	0.24	0.1327
	F_{ST}500	500	2.02	0.18	0.21	0.1398
	Rdm500	500	2.06	0.19	0.22	0.1423
	F_{ST}350+Rdm150	500	2.04	0.19	0.22	0.1373
	F_{ST}250+Rdm250	500	2.05	0.18	0.21	0.1418
	F_{ST}150+Rdm350	500	2.04	0.18	0.21	0.1233
2010 (n=28)	Complete	2983	2.27	0.21	0.24	0.1386
	F_{ST}500	500	1.99	0.18	0.2	0.1326
	Rdm500	500	2.05	0.19	0.21	0.1194
	F_{ST}350+Rdm150	500	1.99	0.18	0.21	0.1343
	F_{ST}250+Rdm250	500	2.01	0.18	0.21	0.1344
	F_{ST}150+Rdm350	500	2.00	0.17	0.20	0.1446
2012 (n=30)	Complete	2983	2.33	0.23	0.24	0.0699
	F_{ST}500	500	2.04	0.20	0.21	0.0435
	Rdm500	500	2.08	0.20	0.22	0.0754
	F_{ST}350+Rdm150	500	2.06	0.20	0.21	0.0641
	F_{ST}250+Rdm250	500	2.06	0.20	0.21	0.0662
	F_{ST}150+Rdm350	500	2.05	0.20	0.21	0.0621
2015 (n=31)	Complete	2983	2.32	0.22	0.24	0.0796
	F_{ST}500	500	2.03	0.19	0.21	0.0805
	Rdm500	500	2.07	0.20	0.21	0.0773
	F_{ST}350+Rdm150	500	2.03	0.19	0.21	0.0813
	F_{ST}250+Rdm250	500	2.02	0.19	0.21	0.0867
	F_{ST}150+Rdm350	500	2.02	0.19	0.20	0.0818
2017 (n=30)	Complete	2983	2.31	0.22	0.24	0.0780
	F_{ST}500	500	2.04	0.19	0.21	0.0736
	Rdm500	500	2.06	0.20	0.21	0.0775
	F_{ST}350+Rdm150	500	2.05	0.20	0.21	0.0750
	F_{ST}250+Rdm250	500	2.06	0.19	0.21	0.0798
	F_{ST}150+Rdm350	500	2.04	0.19	0.21	0.0825
2018 (n=39)	Complete	2983	2.37	0.27	0.26	-0.0581
	F_{ST}500	500	2.11	0.24	0.23	-0.0674
	Rdm500	500	2.14	0.25	0.23	-0.0652
	F_{ST}350+Rdm150	500	2.12	0.25	0.23	-0.0692
	F_{ST}250+Rdm250	500	2.10	0.24	0.22	-0.0659
	F_{ST}150+Rdm350	500	2.11	0.24	0.22	-0.0683

Table S4 – Pairwise F_{ST} estimated between years with the complete dataset (nextRAD_complete) and the five subsets of 500 loci.

		1999	2000	2002	2004	2006	2008	2009	2010	2012	2015	2017
**nextRAD_complete**	**2000**	.0009										
	**2002**	.0016	.0013									
	**2004**	.0019	.0026	.0002								
	**2006**	.0019	.0017	.0006	.0008							
	**2008**	.0017	.0006	.0007	.0011	.0008						
	**2009**	.0019	.0029	.0014	.0020	.0015	.0011					
	**2010**	.0008	.0017	-.0002	0.000	-.0006	.0003	.0002				
	**2012**	.0020	.0020	-.0001	.0004	.0005	.0017	.0012	0			
	**2015**	.0035	.0038	.0019	.0013	.0021	.0019	.0020	.0008	.0018		
	**2017**	.0032	.0034	.0017	.0020	.002	.0025	.0028	.0013	.0011	-.0002	
	**2018**	.0055	.0049	.0032	.0040	.0037	.0031	.0041	.0031	.0032	.0013	.0014
		**1999**	**2000**	**2002**	**2004**	**2006**	**2008**	**2009**	**2010**	**2012**	**2015**	**2017**
**Fst500**	**2000**	.0065										
	**2002**	.0107	.0080									
	**2004**	.0082	.0101	.0090								
	**2006**	.0094	.0082	.0070	.0073							
	**2008**	.0103	.0057	.0080	.0079	.0066						
	**2009**	.0119	.011	.0111	.0125	.0094	.0079					
	**2010**	.0087	.0083	.0060	.0078	.0046	.0074	.0102				
	**2012**	.0098	.0087	.0072	.0096	.0091	.0091	.0103	.0094			
	**2015**	.0127	.0104	.0091	.0086	.0078	.0098	.0102	.0071	.0092		
	**2017**	.0125	.0098	.0106	.0106	.0095	.0105	.0100	.0112	.0097	.0053	
	**2018**	.0142	.0113	.0099	.0124	.0095	.0092	.0125	.0102	.0108	.0062	.0069

Table S4 – Continuation.

		1999	2000	2002	2004	2006	2008	2009	2010	2012	2015	2017
**Rdm500**	**2000**	.0024										
	**2002**	.0028	.0004									
	**2004**	.0020	.0029	-.0002								
	**2006**	.0025	.0024	-.0004	.0000							
	**2008**	.0033	.0012	.0003	.0010	.0017						
	**2009**	.0023	.002	.0016	.0003	.0002	.0000					
	**2010**	.0015	.0024	-.0003	.0006	-.0012	.0006	-.0001				
	**2012**	.0036	.0023	.0000	-.0008	.0002	.0005	.0000	.0002			
	**2015**	.0052	.0035	.0030	-.0005	.0004	.0026	.0037	-.0002	.0025		
	**2017**	.0054	.0040	.0021	.0009	.0019	.0039	.0025	.0012	.0029	-.0010	
	**2018**	.0060	.0038	.0015	.0040	.0012	.0024	.0021	.0022	.0022	.0011	.0019
		**1999**	**2000**	**2002**	**2004**	**2006**	**2008**	**2009**	**2010**	**2012**	**2015**	**2017**
**Fst250+Rdm250**	**2000**	.0043										
	**2002**	.0072	.0046									
	**2004**	.0053	.0071	.0047								
	**2006**	.0067	.0052	.0038	.0034							
	**2008**	.0078	.0034	.0043	.0048	.0037						
	**2009**	.0065	.0072	.0065	.0077	.0053	.0048					
	**2010**	.0049	.0048	.0031	.0044	.0019	.0046	.0066				
	**2012**	.0081	.0071	.0041	.0062	.0067	.006	.0066	.0050			
	**2015**	.0101	.0075	.0066	.0062	.0051	.0078	.0062	.0058	.0067		
	**2017**	.0098	.0074	.0071	.0068	.0065	.0077	.0060	.0080	.0058	.0038	
	**2018**	.0109	.0092	.0076	.0109	.0081	.0082	.0100	.0087	.0081	.0069	.0044

Table S4 – Continuation.

		1999	2000	2002	2004	2006	2008	2009	2010	2012	2015	2017
**Fst350+Rdm150**	**2000**	.0042										
	**2002**	.0078	.0053									
	**2004**	.006	.0073	.0058								
	**2006**	.0085	.0065	.0045	.0048							
	**2008**	.0078	.0044	.0061	.0061	.0054						
	**2009**	.0082	.0082	.0079	.0088	.0070	.0053					
	**2010**	.0048	.006	.0037	.005	.0035	.0051	.0069				
	**2012**	.0082	.0079	.0052	.0076	.0077	.007	.0071	.0067			
	**2015**	.0104	.0084	.007	.0065	.0076	.0094	.0085	.0065	.0065		
	**2017**	.0095	.0079	.0075	.0087	.0068	.0075	.0066	.0081	.0079	.0043	
	**2018**	.0115	.0097	.0077	.0102	.0085	.0081	.0100	.0086	.0089	.0056	.0045
		**1999**	**2000**	**2002**	**2004**	**2006**	**2008**	**2009**	**2010**	**2012**	**2015**	**2017**
**Fst150+Rdm350**	**2000**	.0037										
	**2002**	.0054	.0033									
	**2004**	.0043	.0053	.0042								
	**2006**	.0063	.0051	.0031	.0030							
	**2008**	.0045	.0028	.0028	.0035	.0027						
	**2009**	.0069	.0062	.0046	.0069	.0053	.0040					
	**2010**	.0052	.0033	.0007	.0038	.0013	.0042	.0041				
	**2012**	.0058	.0068	.002	.0057	.0053	.0050	.0057	.0060			
	**2015**	.0087	.0062	.0022	.0054	.0029	.0049	.0044	.0035	.0043		
	**2017**	.0088	.0064	.0042	.0056	.0060	.0059	.0055	.0068	.0051	.0033	
	**2018**	.0094	.008	.0043	.0070	.0062	.0063	.0076	.0059	.0061	.0039	.0038

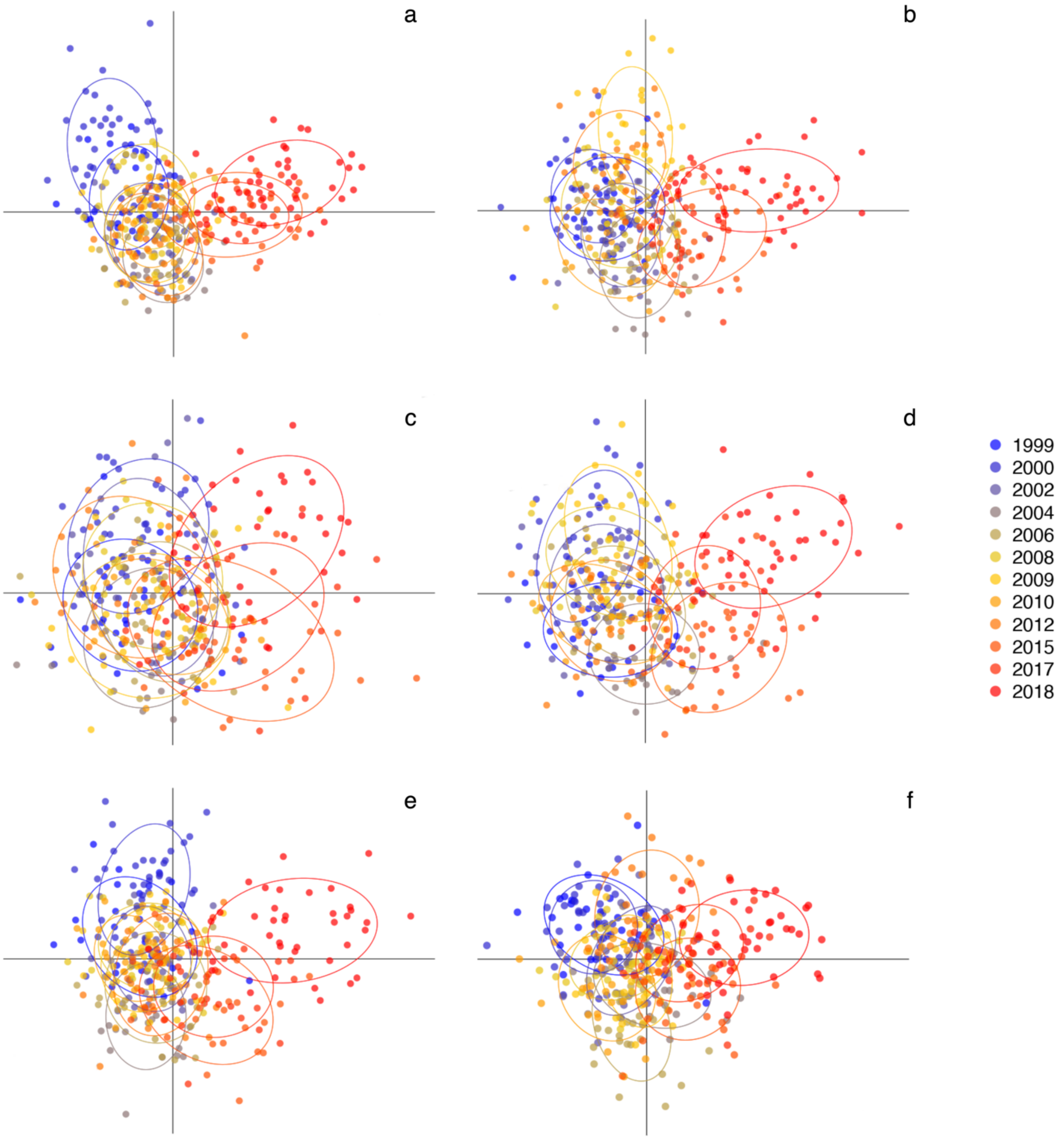

Figure S1 – Discriminant Analysis of Principal Components (DAPC) performed with **a)** complete set of 2,983 microhaplotypes (nextRAD_complete), **b)** subset of 500 loci with higher F_{ST} (Fst500), **c)** subset of 500 randomly selected loci (Rdm500), **d)** subset of 350 loci with higher F_{ST} plus 150 selected at random (Fst350+Rdm150), **e)** 250 loci with higher F_{ST} and 250 selected at random (Fst250+Rdm250), and **f)** subset of 150 loci with higher F_{ST} plus 350 loci randomly selected (Fst150+Rdm350). The subset of 500 loci with the most similar distribution of groups (years) to the nextRAD_complete across the DAPC space was the Fst250+Rdm250 subset (panel e).

Table S5 – Genotypes from the two SNPs in the sex-linked locus HAM6 obtained with the optimized GT-seq panel for 15 samples previously sequenced using Sanger sequencing by Caeiro-Dias et al. (2023) showing 100% assignment consistency between sequencing methods. Corresponding NCBI-SRA BioSample accession numbers are also provided.

Individual	BioSample (SAMN)	1st SNP		2nd SNP		Sex ID
		GT-seq	Sanger-seq	GT-seq	Sanger-seq	
MJO0622_01	54370088	G,T	G,T	A,C	A,C	Male
MJO0622_02	54370089	G,T	G,T	A,C	A,C	Male
MJO0622_04	54370090	G,T	G,T	A,C	A,C	Male
MJO0622_06	54370091	T,T	T,T	C,C	C,C	Female
MJO0622_09	54370092	T,T	T,T	C,C	C,C	Female
MJO0622_10	54370093	T,T	T,T	C,C	-	Female
MJO0622_11	54370094	T,T	T,T	C,C	C,C	Female
MJO0622_12	54370095	G,T	G,T	A,C	A,C	Male
MJO0622_13	54370096	G,T	G,T	A,C	A,C	Male
MJO0622_14	54370097	T,T	T,T	C,C	C,C	Female
MJO0622_15	54370098	G,T	G,T	A,C	A,C	Male
MJO0622_17	54370099	T,T	T,T	C,C	C,C	Female
MJO0622_18	54370100	G,T	G,T	A,C	-	Male
MJO0622_19	54370101	T,T	T,T	C,C	C,C	Female
MJO0622_20	54370102	G,T	G,T	A,C	A,C	Male

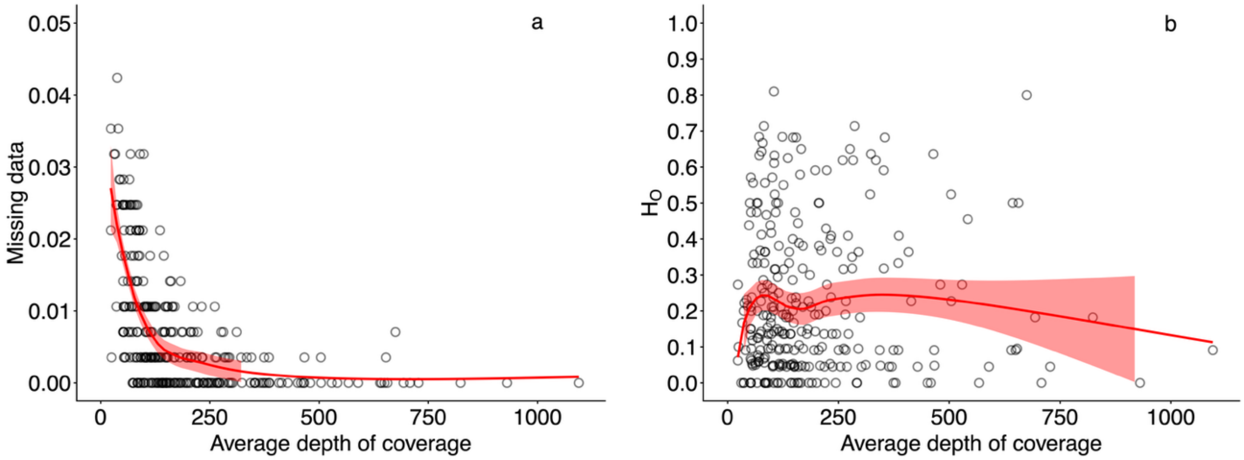

Figure S2 – Effect of depth of coverage on missing data and observed heterozygosity (H_O) in the samples from 1999 genotyped with the GT-seq panel (283 loci) that were used for panel validation. **a)** Relationship between average depth of coverage and missing data. **b)** Relationship between average depth of coverage and observed heterozygosity. Each circle represents one locus; red lines represent the non-linear regressions; and red areas the 95% confidence intervals. Non-linear regressions were fitted with the *loess* method implemented in the *geom_smooth* function from ggplot2 R package v. 4.0.1 (Wickham 2016) performed with R v. 4.4.3 (R Core Team, 2025) in RStudio v. 2024.12.1+563 (RStudio Team, 2025).

References

Barrett, T., Dowle, M., Srinivasan, A., Gorecki, J., Chirico, M., Hocking, T. (2020). data.table: Extension of `data.frame`. R package version 1.13.0. URL:https://CRAN.R-project.org/package=data.table

Bilton, T. P., McEwan, J. C., Clarke, S. M., Brauning, R., van Stijn, T. C., Rowe, S. J., & Dodds, K. G. (2018). Linkage disequilibrium estimation in low coverage high-throughput sequencing data. *Genetics*, **209**, 389–400.

Bilton, T. P. (2019). GUSbase: Genotyping Uncertainty with Sequencing data - Base package. R package version R package version 0.2.0. URL:https://github.com/tpbilton/GUSbase

Bolger, A. M., Lohse, M., & Usadel, B. (2014). Trimmomatic: A flexible trimmer for Illumina sequence data. *Bioinformatics*, **30**, 2114–2120.

Caeiro-Dias, G., Osborne, M. J., Waterman, H. M., Krabbenhoft, T. J., & Turner, T. F. (2023). Limited evidence for extensive genetic differentiation between X and Y chromosomes in *Hybognathus amarus* (Cypriniformes: Leuciscidae). *Journal of Heredity*, **114**, 470–487.

Danecek, P., Auton, A., Abecasis, G., Albers, C. A., Banks, E., DePristo, M. A., Handsaker, R. E., Lunter, G., Marth, G. T., & Sherry, S. T. (2011). The variant call format and VCFtools. *Bioinformatics*, **27**, 2156–2158.

Garrison, E., & Marth, G. (2012). Haplotype-based variant detection from short-read sequencing. *arXiv Preprint arXiv:1207.3907*.

Jombart, T. (2008). adegenet: A R package for the multivariate analysis of genetic markers. *Bioinformatics*, **24**, 1403–1405.

Jombart, T., & Ahmed, I. (2011). adegenet 1.3-1: New tools for the analysis of genome-wide SNP data. *Bioinformatics*, **27**, 3070–3071.

Langmead, B., & Salzberg, S. L. (2012). Fast gapped-read alignment with Bowtie 2. *Nature Methods*, **9**, 357.

Li, H., Handsaker, B., Wysoker, A., Fennell, T., Ruan, J., Homer, N., Marth, G., Abecasis, G., & Durbin, R. (2009). The sequence alignment/map format and SAMtools. *Bioinformatics*, **25**, 2078–2079.

Mangiafico, S. (2020). rcompanion: Functions to Support Extension Education Program Evaluation. version 2.3.21. Rutgers Cooperative Extension. New Brunswick, New Jersey. https://CRAN.R-project.org/package=rcompanion

Osborne, M. J., Caeiro-Dias, G., & Turner, T. F. (2022). Transitioning from microsatellites to SNP-based microhaplotypes in genetic monitoring programmes: Lessons from paired data spanning 20 years. *Molecular Ecology,* **32**, 316–334.

Paradis, E. (2010). pegas: An R package for population genetics with an integrated–modular approach. *Bioinformatics*, **26**, 419–420.

Puritz, J. B., Hollenbeck, C. M., & Gold, J. R. (2014). dDocent: A RADseq, variant-calling pipeline designed for population genomics of non-model organisms. *PeerJ*, **2**, e431.

R Core Team (2019). R: A language and environment for statistical computing. R Foundation for Statistical Computing, Vienna, Austria. URL https://www.R-project.org/.

R Core Team (2025). R: A language and environment for statistical computing. R Foundation for Statistical Computing, Vienna, Austria. URL https://www.R-project.org/.

RStudio Team (2019). RStudio: Integrated Development Environment for R. Posit Software, PBC, Boston, MA. URL http://www.posit.co/.

RStudio Team (2025). RStudio: Integrated Development Environment for R. Posit Software, PBC, Boston, MA. URL http://www.posit.co/.

Quinlan, A. R., & Hall, I. M. (2010). BEDTools: A flexible suite of utilities for comparing genomic features. *Bioinformatics*, **26**, 841–842.

Wickham H. (2016). ggplot2: Elegant Graphics for Data Analysis. R package version 4.0.1. URL https://ggplot2.tidyverse.org

Wickham, H. (2019). stringr: Simple, Consistent Wrappers for Common String Operations. R package version 1.4.0. URL https://CRAN.R-project.org/package=stringr.

Wickham, H., François, R., Henry L., Müller, K., Vaughan, D., (2020). dplyr: A Grammar of Data Manipulation. R package version 1.0.0. URL https://CRAN.R-project.org/package=dplyr.

Willis, S. C., Hollenbeck, C. M., Puritz, J. B., Gold, J. R., & Portnoy, D. S. (2017). Haplotyping RAD loci: An efficient method to filter paralogs and account for physical linkage. *Molecular Ecology Resources*, **17**, 955–965.
